# Bone Morphogenetic Protein 4 Signalling in Neural Stem and Progenitor Cells during Development and after Injury

**DOI:** 10.1155/2016/9260592

**Published:** 2016-05-16

**Authors:** Alistair E. Cole, Simon S. Murray, Junhua Xiao

**Affiliations:** Department of Anatomy and Neuroscience, School of Biomedical Sciences, Faculty of Medicine, Dentistry & Health Sciences, The University of Melbourne, Parkville, VIC 3010, Australia

## Abstract

Substantial progress has been made in identifying the extracellular signalling pathways that regulate neural stem and precursor cell biology in the central nervous system (CNS). The bone morphogenetic proteins (BMPs), in particular BMP4, are key players regulating neuronal and glial cell development from neural precursor cells in the embryonic, postnatal, and injured CNS. Here we review recent studies on BMP4 signalling in the generation of neurons, astrocytes, and oligodendroglial cells in the CNS. We also discuss putative mechanisms that BMP4 may utilise to influence glial cell development following CNS injury and highlight some questions for further research.

## 1. Introduction

Neural stem cells (NSCs) are self-renewing, multipotent progenitor cells that can generate neurons as well as the two major glial cell types, oligodendrocytes and astrocytes [1, 2]. Altman and Das first speculated about the possibility of postnatal neurogenesis generated by an unidentified pool of undifferentiated cells located around the ventricular and germinal zones in young rats [3]. This has since been attributed to NSCs migrating from stem cell niches located in the subventricular zone (SVZ, also known as the subependymal zone) [4]. Adult NSCs share common features with astrocytes [5], and can be identified by nestin, glial fibrillary acidic protein (GFAP), and Sox2 expression [6, 7]. Adult NSCs are derived from embryonic radial glia-like cells (RGCs) during development [8] and are specified at approximately E11.5 in murine embryogenesis [9]. Adult NSCs can give rise to neural precursor cells (NPCs), which include neuroblasts [10] and glial precursor cells such as oligodendrocyte progenitor cells (OPCs) [11, 12]. Neurogenesis also occurs in the hippocampal subgranular zone (SGZ) of the dentate gyrus from precursor cells with stem-like properties. Whether or not these SGZ progenitor cells are “true” stem cells has been debated [13, 14]. There is evidence that they do not self-renew indefinitely but can give rise to all neuronal subtypes through sequential differentiation [13, 15]. These two regions are currently the only known source of NSCs in the mammalian brain [2, 14].

Several key signalling pathways govern the regulation of NSC maintenance and specification in the adult CNS. These include WNT/*β*-catenin [16], Sonic hedgehog (Shh) [17, 18], fibroblast growth factor (FGF) [19], and bone morphogenetic protein (BMP) signalling [20], with degrees of crosstalk between many of these pathways [16, 21–23]. This review will examine the role of BMP signalling in NSC specification in the developing, adult, and injured CNS. In particular, it will focus on the role of BMP4, which has a particularly well-characterised effect on glial development [24]. SVZ NSCs have been better characterised in regard to BMP4 signalling compared to SGZ NSCs [20] and will be discussed in this review at the expense of the latter.

## 2. BMP4 Signalling Is a Complex, Tightly Regulated System

BMPs are the largest class in the transforming growth factor *β* (TGF-*β*) superfamily, with at least 20 structurally distinct members. Aside from their eponymous functions in bone and cartilage formation, they also have defined roles in cellular and developmental processes including proliferation and differentiation, cell-fate determination, and apoptosis [25]. A protein preparation contributing to osteogenesis was first isolated from decalcified bone extracts and studied for its stimulating effect on chondrocytes, osteoblasts, and osteoclasts by Urist in 1965 [26]. It was initially unclear as to whether a single protein within this mixture was responsible, but subsequent studies by Urist and others lead to the characterisation of several proteins described as “bone morphogenetic proteins” due to their critical role in bone formation [27, 28]. Their contribution to vertebrate development has since been shown to be so extensive that several researchers have suggested that the name “body morphogenetic proteins” would better describe their significance [29, 30]. Within this broad and heterogeneous family, BMP4 in particular has many critical roles in the development of the nervous system during embryogenesis [20]. Furthermore, BMP4 reemerges as an important factor regulating neural cell fate determination during adulthood and following CNS injury.

BMP4 was purified and cloned by Wozney et al. in 1988 and was originally known as BMP-2B due to its DNA sequence similarity to BMP2 [31]. Structurally, human BMP4 is a highly conserved, 116-residue protein that is posttranslationally cleaved from a 408-residue preproprotein. The functional BMP4 peptide chain (from residues 292 onwards) is highly conserved between human, mouse, rat, and zebrafish [32]. The C-terminus contains seven conserved cysteine residues that are glycosylated, leading to the formation of a characteristic cysteine knot structure; this domain allows BMP4 to assemble into a biologically active homodimer and form heterodimers with other BMPs [33].

After synthesis at the endoplasmic reticulum and posttranslational modifications in the Golgi apparatus, the BMP4 peptide chain is proteolytically cleaved and dimerization occurs at the Mad homology (MH2) domain. BMP4 also has a unique secondary cleavage site that governs whether it is subsequently secreted as a short, soluble isoform or longer isoform that is tethered to the extracellular matrix (ECM) [34, 35]. Thus, BMP4 can have local or regional effects depending on cleavage of this secondary prodomain, the exact mechanisms of which remain unclear and are likely to be context-dependent [29]. It can also be carried via matrix vesicular transport, although the exact isoform of BMP4 transported remains unknown [36].

Given the variety of cell types and tissues that it influences, the BMP signalling network is a fittingly diverse affair. BMP4 signalling is transduced through the canonical TGF-*β* family pathway [37–40]. This involves glycosylated BMP4 forming homodimers in the extracellular space or extracellular matrix and subsequent binding to a membrane-bound receptor complex. This complex is classically comprised of two BMP Type I serine-threonine kinase receptors, of which there are two classes, BMPRIA (or ALK3) and BMPRIB (or ALK6), and two of a single class of Type II receptor, BMPRII. All three receptors contain two conserved functional domains flanking a typical transmembrane domain: an N-terminal extracellular ligand-binding domain for BMP homodimer interaction and a C-terminal intracellular kinase domain. Structurally similar receptors may also act as receptors for BMP4. Activin Receptor Type 1 (ACVR1) can act as a Type I receptor for BMP4 under certain contexts [41]. Similarly, Activin Receptor Type II (ActRII) and Activin Receptor Type IIB (ActRIIB) can act as Type II receptors, with similar binding affinities for BMP4 in certain tissues [42, 43].

**Figure 1:**
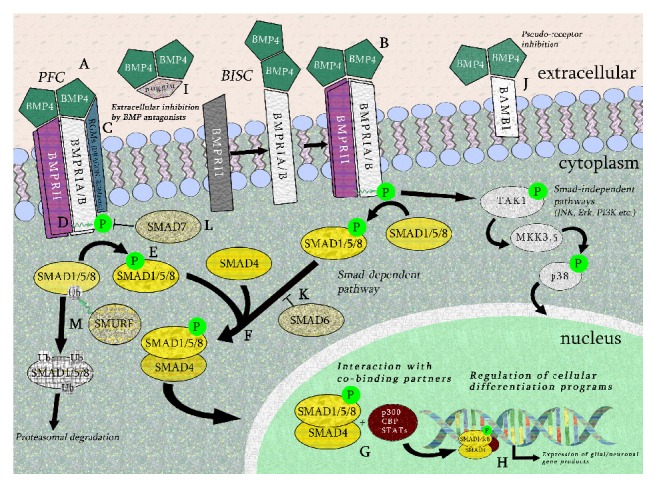
General BMP4 cellular signalling pathway. BMP4 dimers may bind to preformed complexes (PFCs, A), in which BMPRI Type I and Type II receptors are already bound at the cell surface, or by firstly binding to the Type I receptor and inducing the Type II receptor to the complex (BMP-induced signalling complex or BISC, B). Repulsive guidance molecules (RGMa, DRAGON, etc.) may enhance binding of the BMP4 dimer to the Type I receptors in both PFC and BISC binding (C). In the canonical SMAD-dependent pathway, the constitutively active Type II receptor kinase domain phosphorylates a glycine-serine-rich area known as the “GS box” on the Type I receptor (D). The activated Type I receptor sequentially phosphorylates receptor-associated SMADs (SMAD1, SMAD5, and SMAD8 in the case of BMP4) (E). These receptor-associated SMADs then form complexes with Co-SMAD4 (F), enter the nucleus, and further associate with cobinding partners including p300, CBP, STATs, and others (G). These heteromeric complexes then act as transcription factors to regulate the expression of neuronal and glial gene products (H). Extracellular inhibitors of the BMP4, such as noggin, bind BMP4 prior to receptor binding (I). Pseudoreceptors such as BAMBI bind BMP4 dimers but do not propagate downstream signalling activity to a lack of an intracellular kinase domain (J). Other inhibitory intracellular factors include SMAD6, SMAD7, and SMURF. SMAD6 competes with SMAD4 for binding to receptor-associated SMADs (K), SMAD7 blocks the kinase domain of BMP Type I receptors (L), and SMURF mediates ubiquitination and subsequent proteasomal degradation of receptor SMAD1, SMAD5, and SMAD8 (M). Other SMAD-independent pathways may be activated by BMP4, such as MAPK/p38, JNK, Erk, and PI3K (MAPK/p38 pathway shown in this figure).

Signalling may occur through two mechanisms: preformed complexes (PFCs) of Type I/Type II receptors binding to BMP4 homodimers or initial binding of BMP4 homodimers to the high affinity Type I receptor, which then recruits the Type II receptor to the complex (BMP-Induced Signal Complex or BISC) [44]. Comparatively, BISC signalling is reliant upon cholesterol-enriched regions of the plasma membrane to facilitate BISC formation, whereas PFC signalling does not. However, PFC signalling does appear to require clathrin-mediated endocytosis of the receptor complex to transmit downstream signalling [45]. In general, BMP4 has much higher affinity for its Type I receptors than the Type II receptor [46–49]; direct binding to the Type II receptor is less common. In the canonical BMP signalling pathway, upon binding of the BMP4 homodimer to the receptor complex, conformational changes allow the constitutively active Type II receptor to phosphorylate a conserved glycine/serine box on the Type I receptor kinase domain. This activated Type I receptor then propagates the signal downstream by phosphorylation of the SMAD (signalling mothers against decapentaplegic [50]) family of intracellular signalling molecules (see Figure 1).

BMP4 signalling through complexes comprised of BMPRA/IB and BMPRII preferentially phosphorylates receptor-associated SMAD1, SMAD5, and SMAD8 (known as the R-SMADs) [29], as opposed to SMAD2 and SMAD3. These activated R-SMADs can each form heteromeric complexes with Co-SMAD4, which translocates to the nucleus and acts as a transcription factor (TF), binding cooperatively with other TFs and interacting with specific regulatory DNA sequences to control gene expression [51, 52]. In certain contexts, activated BMPRIA/B may also signal through the p38/mitogen-activated protein kinase (MAPK) pathway in a SMAD-independent manner [53]. Other SMAD-independent or noncanonical BMP signalling pathways have been documented in various applications [54]: these will be selectively discussed as they pertain to neural stem and precursor cell differentiation.

Precise spatiotemporal regulation of BMP signalling is vital due to the many roles that BMPs exert during development and adulthood in multiple tissue-specific processes. As such, the BMP4 signalling pathway can be regulated by numerous extracellular and intracellular factors. Several endogenous extracellular inhibitors of BMP4 have been classified, including noggin [55], chordin [56], FSTL1 [57], DAN (NBL1) [58], and gremlin [59] (for review, see Mulloy and Rider, 2015 [60]). Secretion of noggin, follistatin, and chordin by specialised groups of cells known as organisers is particularly crucial during development to balance the dorsalising effects of BMPs during gastrulation [20, 49]. At a receptor-ligand level, several receptor cobinding partners can enhance or inhibit BMP4 homodimer ligand binding to regulate downstream signalling. For example, BAMBI (BMP and activing membrane-bound inhibitor) is a BMP receptor analogue with similar extracellular protein binding sites as the Type I receptors, but lacking a concomitant kinase domain. This pseudoreceptor competitively binds BMP4 homodimers but prevents further downstream phosphorylation [61]. Another factor, DRAGON, is a glycosylphosphatidylinositol-anchored protein from the repulsive guidance molecule (RGM) family. This protein associates with both types of receptors at the external cell membrane and binds directly to BMP4, enhancing its binding to the receptor complex [62]. A DRAGON homologue, repulsive guidance molecule A (RGMa) enhances binding of BMP2 and BMP4 to BMP Type I receptors, leading to activation of BMP-SMAD signalling [63]. Both DRAGON and RGMa are expressed in the murine neural tube during embryogenesis [62, 63], corresponding to the increased role of BMP-SMAD signalling during this process [64].

At an intracellular level, inhibitory SMAD7 is a cytosolic factor that stably binds to the activated Type I receptors [65] and prevents R-SMADs from being phosphorylated. Downstream of BMP ligand-receptor interactions, SMAD molecules themselves are also subject to regulation. Inhibitory SMAD6 competitively binds with SMAD4 to disrupt the formation of the R-SMAD/SMAD4 TF complex [66]. SMAD specific E3 ubiquitin protein ligases (also known as Smad Ubiquitin Regulatory Factor or SMURF) 1 and 2 are factors that modulate levels of ubiquitinated cytosolic R-SMADs [67]. They may also cooperatively bind with inhibitory SMADs such as SMAD7 to target receptor degradation [68].

Heterodimerisation of BMPs is another extracellular method of signalling regulation [29, 69, 70]. The shared cysteine knot domain allows BMP members to form heterodimers with other BMPs [71]. For instance, BMP4 may bind with BMP7 to form a BMP4-BMP7 heterodimer; this is thought to promote more effective signal transduction in certain applications [72]. The degradation pathway of the BMP4 homodimer and other heterodimers after binding remains unclear.

The specificity of downstream transcriptional control of gene expression by BMP4 is largely dependent on the particular ligand-receptor combinations at the cellular membrane [29]. Several factors can contribute to this interaction: the composition of BMP dimer ligand, whether signalling occurs through the PFC model or BISC model, and the relative contributions of the two different Type I receptors in mediating downstream activity. In particular, variable signalling through BMPRIA/BMPRII complexes versus BMPRIB/BMPRII complexes has been shown to occur at different stages in development, affecting unique downstream targets and regulating diverse cellular processes [38, 73]. Studies using fibroblastic, myoblastic, and osteoblastic cell lines have also shown that BMP2 signalling occurring through PFC complexes preferentially activates SMAD-dependent pathways, whereas BISC binding activates non-SMAD pathways [44, 53]. It is not known whether preferential signalling activation occurs in neural stem and progenitor cells. It should be noted that studies on interactions between ligand-receptor complexes and uncharacterised proteins are still informing the field on novel BMP signalling mechanisms. For example, recent proteomic analysis of novel regulators of BMP signalling has identified for the first time a non-SMAD protein (protein associated with SMAD1, or PAWS1) that can act as a substrate for BMPRIA phosphorylation. Subsequent interaction with SMAD1 leads to the upregulation of SMAD4-independent target genes, as well as possible novel interactions beyond the canonical BMP signalling pathway [74].

## 3. BMP4 Signalling in Neural Stem and Precursor Cells during Embryonic Development

Since their discovery as osteoinductive factors, BMPs have also been shown to play a crucial role in the development of the nervous system, specifically neuroectoderm induction, neural crest cell specification, and CNS neural patterning [20, 64, 75]. BMP4 in particular has been shown to be critical during early murine embryonic development:* Bmp4* deletion in mice is lethal 6.5 to 9.5 days postcoitum [76]. Deletion of its cognate receptor BMPRIA is also embryonic-lethal in mice [77]. During early embryonic development, expression of BMPs is actively inhibited by secretion of noggin, chordin, and follistatin from embryonic organisers to allow neural induction to commence [78].* In vitro* application of noggin to human embryonic stem cells activates microRNA-mediated degradation of SMAD4 transcripts. This mechanism putatively acts* in vivo* to block BMP4-SMAD signalling pathways during neural induction [79]. Repression or activation of BMP signalling, in conjunction with a corresponding gradient of Sonic Hedgehog (Shh) expression, actively specifies the ectoderm into neuronal or nonneuronal tissue, respectively. Additionally, other signalling pathways that generally antagonise BMP signalling, such as fibroblast growth factor (FGF) [19] and Insulin-like Growth Factor-1 (IGF1) [80], play a role in modulating the levels of active BMP signalling at this stage, by downregulating both BMP4 expression and phospho-SMAD activation by BMPRIA/B. This complementary morphogenic gradient of BMPs (including BMP4 and others including BMP2 and Growth Differentiation Factor 7 (GDF7)) and Shh signalling establishes the dorsoventral axis, with the area of intermediate signalling specifying the neural crest cells (NCCs) that eventually form the peripheral, sympathetic, and sensory nervous systems [81–83]. NCC specification occurs in conjunction with two pathways strongly associated with BMP: WNT/*β*-catenin [84, 85] and Notch signalling [86]. WNT/*β*-catenin is a particularly frequent collaborator with BMP4, with temporally and spatially similar actions in development and adulthood [87, 88].

Following neural induction, secretion of BMP4 from ectoderm and neural tube roofplate cells promotes subsequent neural patterning of several key CNS topographies, including forebrain, cerebellum, and dorsal spinal cord. Again, the dualistic relationship between BMP4 and Shh signalling from the notochord and floorplate is important for dorsoventral axis development in the spinal cord. Liem et al. showed that a dorsal cellular identity does not occur by default due to lack of the Shh ventralising signal. Rather, they showed that the dorsalising signal provided by BMP4 (and 7) to early neural tissue explants directly induces the expression of high levels of definitive dorsal cell markers MSX1, PAX3, DSL1, and SLUG in these cells [89].

Further to this finding, Wine-Lee et al. showed that ablation of BMP Type I receptors BMPRIA/B from the neural tube disrupts proper dorsal-ventral interneuron specification [90], with BMPRIA/B overexpression in the chick spinal cord causing dorsalisation to occur in ventral spinal cord regions [91]. Interactions of BMP4 with WNT/*β*-catenin are crucially important at this stage. Shortly following neural crest formation, contemporaneous WNT/*β*-catenin signalling downstream of BMP4 coordinates transcriptional control of the neurogenin-1 (Ngn1) and Olig3 neuron-specific TFs [92, 93], which are crucial for dorsal interneuron specification. Ille et al. have shown that this involves a balance of the proliferation-inducing WNT effect against the differentiation-promoting BMP4 effect on NSCs. This balance may be required to maintain a population of cycling, dorsal interneuron progenitors during spinal cord development [16]. In addition to spinal cord patterning, BMP4 has also been implicated in proper forebrain development [94], as well as early postnatal cerebellar cell differentiation [95].

As well as these early effects in neural induction, neural crest specification, and dorsoventral patterning, BMPs have a significant, temporally dependent influence on both neuronal and glial differentiation of embryonic NSCs and NPCs. Following gastrulation, BMP4 signalling specifies NSCs and NPCs towards neuronal lineage commitment in both the CNS and PNS [64]. In the CNS, Li et al. showed that treatment of embryonic day 13 (E13) rat neocortical neuroepithelial cells with BMP4* in vitro* significantly increased expression of neural markers MAP-2 and TUJ1 and resulted in longer neurite outgrowth. This was mediated through BMPRIA, as truncated forms of the receptor significantly reduced this effect [96]. The capacity of BMPRIA mutant neurites to respond to brain-derived neurotrophic factor (BDNF) was preserved, suggesting that this was not a blockage of nonspecific differentiation factors. Further investigation using bioinformatic techniques would be helpful to rule out the possibility of the truncated receptor blocking other signalling pathways beyond BMP signalling.

The neurogenic effect of BMP4 during forebrain cortical neurogenesis has been associated with the SMAD-independent MAPK/extracellular signal-related kinase (ERK) pathway. Moon et al. used primary cerebral cortical stem cells from E13.5 rats to demonstrate that BMP4 exposure promotes expression of TUJ1 through MAPK/ERK activation. This was further linked to signal crosstalk between BMP4 and WNT/*β*-catenin, with a WNT/*β*-catenin signalling activator increasing subsequent downstream BMP4 mRNA transcription. Increased BMP4 levels promote Ras-mediated ERK signalling cascade activation. This occurs synergistically with suppression of epidermal growth factor receptor (EGFR) signalling by BMP4, simultaneously arresting the mitotic effect of EGF [21] on NSCs and allowing ERK to activate the TUJ1 promoter to induce neuronal differentiation [97]. Here, the complexity of neurogenic signalling regulation is clearly displayed, with BMP4 a key mediator in this major cell signalling network.

## 4. BMP4 Promotes Astrocytic Differentiation through Multiple Mechanisms

As neurogenesis nears completion in late embryonic/early postnatal development, the neurogenic effect of BMP4 is subdued and its dual function as a promoter of astrogliogenesis and inhibitor of oligodendrogliogenesis becomes more pronounced. Several studies from the laboratory of Jack Kessler in the mid-to-late 1990s revealed that BMP4 (as well as related members BMP2 and BMP7) promotes astroglial phenotypes in embryonic neural progenitor cells at late embryonic/perinatal stages. Gross et al. cultured mouse embryonic (E17) multipotent nestin+ neural progenitors from the subventricular zone with exogenous BMPs, including BMP4, and found that BMP treatment significantly increased GFAP+ cells at several time points. They showed that these cells expressed the relevant BMPRIA/B/II receptors to mediate BMP signalling, but no measurements of downstream signalling molecules (e.g., phospho-SMAD1/SMAD5/SMAD8) were attempted [98]. Subsequent studies showed dose-dependent effects of BMP4 on embryonic stem cells* in vitro*, with different concentrations potentially activating different signalling responses and outcomes [99].

The signalling mechanisms by which BMPs promote astrogliogenesis were subsequently revealed by Nakashima et al. to occur through SMAD1 activation and subsequent association with signal transducers and activators of transcription (STATs). STATs are cytoplasmic transcription factors that have a crucial role in relaying signals from the cell membrane to the nucleus. Activated SMAD1/STAT3 form complexes with p300 and CBP (CREB-binding protein), which are multifunctional coactivators that facilitate binding of SMAD1/STAT3 to astrocytic promoters [100]. Rajan et al. further demonstrated that BMP4-mediated astrocytic differentiation also occurs through the interactions of FKBP12/rapamycin associated protein (FRAP) with BMPRIA. FRAP is activated by the serine-threonine kinase FKBP12, which is normally tethered to the inactive BMPRIA receptor. Upon BMP4-induced conformational changes to BMPRIA, FKBP12 is released and associates with FRAP to activate STAT3 [52]. STAT3 then associates with p300/CBP to activate astrocytic gene promoters as described above.

Prior to this, Bonni et al. and others had shown that ciliary neurotrophic factor (CNTF) and leukaemia inhibitory factor (LIF) can promote astrogliogenic specification from NPCs [101, 102]. These pathways synergise with BMP4 signalling-regulated STAT3 activation to promote astrogliogenesis through further activation of STATs via Janus kinases (JaK) [103, 104]. These pathways are not redundant: LIF signalling appears to be important for production of GFAP+ astrocyte progenitors, with BMP4-induced astrocytes producing a more mature, lineage restricted astrocyte morphology [105].

In addition to these mechanisms, p57kip2 is an important upstream promoter of the BMP4-mediated astrogliogenesis and regulates expression of BMP4 antagonists. Short-hairpin RNA suppression of p57kip2 abrogated the typical increase in GFAP+ cells generated from SVZ and SGZ NSCs by exogenous BMP4* in vitro*. They further showed that noggin and chordin expression was increased upon p57kip2 suppression, suggesting possible regulation of expression of these BMP4 antagonists by p57kip2 [106].

## 5. The Specific BMP4 Effect on Embryonic NSCs Is Temporally Controlled

Given the dual role of BMP4 as both a neurogenic (early embryonic) and astrogliogenic (late embryonic/early postnatal) factor in NSCs, precise temporal control of BMP4 activity by extrinsic factors is crucial. For example, Ngn1 is a critical regulator of neurogenesis [107] with high levels of protein and mRNA transcript expression during neurogenesis (~E12.5 to E15.5) but reduced levels during gliogenesis (~P0–P4) [108]. Sun et al. showed that Ngn1 suppresses BMP4-induced astrogliogenesis in E13.5 cortical NSC cultures by sequestering SMAD1/STAT complexes and blocking their interaction with coactivators p300/CBP. This disrupts the activation of astrocytic promoters such as GFAP by these TF complexes. As neurogenesis nears completion, downregulation of Ngn1 expression by mature neuronal cells releases these astrogliogenic transcriptional promoters from sequestration [109]. Further investigation of this effect by Zhao et al. suggests that Ngn1 also increases transcription of microRNA miR-9 to downregulate JaK-STAT1-mediated astrogliogenesis in embryonic stem cell cultures [110]. Thus, the specific effect exerted by BMP4 on NSCs may depend on levels of temporally dependent external regulators, such as Ngn1.

Evidence from chick embryo studies suggests that differential expression of the Type I receptors may also play a part in regulating the dual neurogenic-astrogliogenic effect of BMP4. Using chick explant cultures, Agius et al. observed that NPCs migrated from the neuroepithelium in the mantle layer from E5 (corresponding to a period of neurogenesis in the developing chick embryo) and that GFAP+ astrocytes were not generated in the dorsal neuroepithelium. They demonstrated* in vitro* that these dorsally derived progenitors were amenable to astroglial lineage commitment, but that increased BMP4-SMAD signalling through BMPRIB in the dorsal-most regions of the neuroepithelium promoted neuronal specification. BMP4 treatment, presumably acting through BMPRIB, completely prevented astrocyte development from more ventrally derived chick spinal cord explants at E5. However, at E6, when BMPRIA is significantly upregulated, astrocyte development was permitted. Noggin treatment at E5 permitted copious GFAP+ expression from dorsal neuroepithelial neural progenitors, suggesting this restriction was an effect of blocking dorsal BMP4-BMPRIB signalling. Thus, the astrogliogenic effect may be due to sudden upregulation of BMPRIA, when at E5 the neurogenic effect of BMP4 is mediated through BMPRIB [111]. This meshes well with evidence describing a direct role of BMPRIA in promoting astrogliogenesis in a SMAD-independent manner through FRAP activation of STATs [52]. Replicating these findings in transgenic mouse models allowing selective ablation of these receptors is crucial to further clarify specific temporal functions of BMPRIA/B during neuronal development.

## 6. BMP4 Is Critical for Suppression of Oligodendrogliogenesis

Perhaps the most well-characterised effect of BMP4 is its inhibitory effect on the myelin-forming oligodendrocyte lineage cells* in vitro* and* in vivo* [98, 112–115]. OPCs are specified from NSCs during development and adulthood and can differentiate into the myelin-forming oligodendrocytes of the CNS [116]. OPCs can also form astrocytes and, in special cases, neurons, leading some to consider the OPC to be more aptly described as an adult NSC [117]. The exact region OPCs are originally derived from was a contentious topic for many years, but recent evidence has determined that embryonic stage OPCs are firstly specified in the ventral ventricular zone of the spinal cord [118]. Specification of these ventrally derived oligodendrocyte-lineage cells as OPCs has been shown to be influenced by Shh signalling from the floorplate and notochord [17, 119].

OPCs also arise from dorsal sources at a later stage in development and are influenced by a Shh-independent pathway [120]. BMP4 has also been shown to inhibit the generation of these dorsally derived OPCs [121]. Developmentally, OPCs are generally characterised by the expression of several markers including the key basic helix-loop-helix (bHLH) transcription factor Olig2, a critical factor that promotes oligodendrocyte lineage commitment [122], and others such as platelet-derived growth factor receptor-*α* (PDGFR*α*) [123], chondroitin sulphate proteoglycan NG2 [124], and the monoclonal antibody O4 [125].

Several groups have shown that excess exogenous BMP4 during development reduces subsequent oligodendrogliogenesis in both the mouse [112] and chick embryo [113]. Mekki-Dauriac et al. also showed that disruption of endogenous BMP4 signalling by transplanting noggin-overexpressing cells produced early dorsal oligodendrocyte production [113]. The effect of BMP4 on OPCs has been shown to be dose-dependent. Grinspan et al. exposed OPCs and “pre-OPCs” (Nestin−/Olig2+ cells lacking classical OPC marker expression) to increasing concentrations of BMP4, with diminishing effects on maturation as the progenitor cells progress through the oligodendrocyte lineage [114].

Given that global genetic knockout of BMP4 and its receptors is embryonic-lethal, conditional genetic ablation driven by expression of temporal markers offers a more nuanced approach to understanding BMP4 signalling in embryonic development. Genetic manipulation of the BMPRI receptors has provided interesting and somewhat counterintuitive insights into the role of BMP4 receptors in specifying OPCs from NSCs during development. Two studies in particular have looked at disruption of BMP4 signalling through deletion of the BMPRIA/B receptors. See et al. used Cre-*loxP*-mediated transgenic excision of the* Bmpr1a* gene from cells expressing BRN4, a broad neural TF activated in early embryogenesis. This was crossed with a conventional* Bmpr1b* KO mouse to generate* Bmpr1a-Bmpr1b* double KO mice. This modification leads to several developmental defects in mice at P0. While numbers of astrocytes in the spinal cord are decreased at P0 compared to controls, disrupted BMP4 signalling through BMPRIA/B does not appear to affect total numbers of OPCs. Intriguingly, the numbers of mature oligodendrocytes expressing common myelin proteins including myelin basic protein (MBP) were reduced at P0. This suggests that some basal level of embryonic BMP signalling through BMPRIA/B is required for prenatal oligodendrocyte maturation [126]. The second study by Samanta et al. deleted BMPRIA only from NPCs expressing Olig1 in the neural tube from E13.5. This did not affect subsequent OPC numbers at birth or P20 [127]. However, at P20, there was an increase in mature oligodendrocytes in the BMPRIA KO group. This study did not discount the possibility of increased compensatory signalling through BMPRIB, as phospho-SMAD1, phospho-SMAD5, and phospho-SMAD8 were still detected.

A further study utilising the* Bmpr1a* conditional KO system examined the role of deleting BMPRIA in* Emx-1* expressing NSCs of the murine telencephalon. It was found that subsequent astrocytes derived from these NSCs aberrantly expressed vascular endothelial growth factor (VEGF) at P10, leading to the disruption of cerebrovascular angiogenesis as well as impaired blood-brain barrier formation [128]. Interestingly, while previous studies using Olig1-*Cre*-driven* Bmpr1a* deletion showed increases in mature O4+ oligodendrocytes at P20, no differences in O4+ cells were observed at P20 in this study. In addition, compared to the earlier study deleting both* Bmpr1a* and* Bmpr1b* from BRN4-expressing cells in which GFAP+ astrocytes are reduced, no such decreases were observed here. These observations can be attributed to the different regional and temporal expression profiles of the Cre promoter driving the deletion of* Bmpr1a*, respectively.

In summary, embryonic studies utilising broad overexpression or inhibition of BMP4 during, or immediately prior to, gliogenesis demonstrate that this decreases or increases subsequent oligodendrogliogenesis, respectively. However, blocking BMP4-SMAD signalling through deletion of BMPRIA/B from early embryonic embryogenesis (prior to OPC specification) reduces numbers of mature oligodendrocytes at P0. Importantly, this is not due to reduction in the numbers of OPCs specified, as no changes in OPC number were detected. Additionally, a second line of inquiry found that reduction, but not complete suppression, of BMP4 signalling through BMPRIA deletion at E13.5 has no effect on OPC numbers at P0. However, by this stage, reduced BMP signalling increases mature oligodendrocyte number by P20. The reasons for this remain unclear. Conclusions from both studies and others suggest that BMP signalling through BMPRIA/BMPRIB does not play a role in specification of OPCs from NSCs but has a strong negative effect on subsequent OPC differentiation [129]. It is likely from the current data that signalling through particular combinations of receptors (e.g., BMPRIA-BMPRII versus BMPRIB-BMPRII complexes) could have unique effects on oligodendrogliogenesis from NSCs and NPCs. Moreover, as described above, the specific regional and temporal expression of particular BMP receptors during development must be considered. Further research using inducible, cell-specific genetic knockouts and pharmacological inhibition of individual BMP receptors could potentially elucidate these mechanisms.

The mechanisms by which BMP4 is thought to modulate oligodendroglial lineage commitment are thought to involve the basic helix-loop-helix transcription factors named “inhibitors of differentiation” or IDs, which are known to be a key downstream target of the BMP/SMAD signalling pathway [130]. Overexpression of ID4 in OPC cultures promotes astrogliogenesis and mimics the effect of BMP4 [131]. Samanta and Kessler cultured neural progenitor cells with BMP4 and a microarray analysis showed that, within the culture, the ID family of transcription factors was significantly upregulated, particularly ID4. Both ID2 and ID4 were then used in a lentivirus overexpression assay using cultured neural progenitor cells. The ID4 group showed a marked decrease in the number of oligodendrocytes, while the number of astrocytes increased 2.5-fold. Mechanistically, coimmunoprecipitation studies showed that the ID proteins inhibited differentiation by complexing with Olig1/2 and preventing them from entering the nucleus. Immunohistochemical analysis showed that, in the absence of BMP4, the Olig transcription factors were localised predominantly in the nucleus. However, in the BMP4 treated group, they were found to be colocalised in the cytoplasm with ID proteins [132].

**Figure 2:**
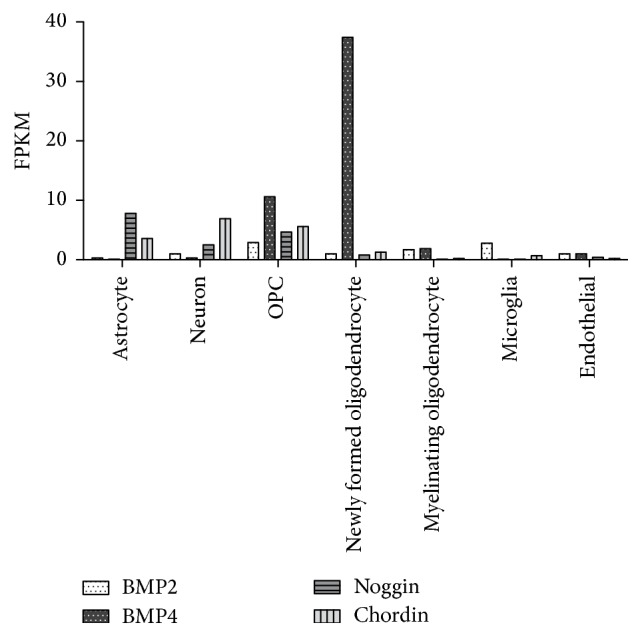
RNA-Seq transcriptome analysis showing increased transcription of* Bmp4* transcripts in OPCs and newly formed oligodendrocytes in postnatal mice compared to other CNS cells. This is unique to* Bmp4*; closely related BMP2 does not show similar levels of increased transcription in oligodendroglial lineage cells compared to other CNS cells. Additionally, increased* Bmp4* transcription does not appear to be counteracted by concomitant transcriptional increases expression of BMP inhibitors, such as noggin and chordin. The functional relevance of increased* Bmp4* transcription by OPCs and immature oligodendrocytes remains to be clarified (FPKM = fragments per kilobase of transcript per million mapped reads.).

A recent RNA sequencing (RNA-Seq) transcriptome database constructed by Zhang et al. examined whole cell gene transcription in multiple CNS cell types in postnatal mice [133]. Interestingly,* Bmp4* shows a tenfold increase in transcription by OPCs compared to astrocytes, neurons, microglia, and endothelial cells at P7. Furthermore, newly formed oligodendrocytes that are not expressing myelin proteins such as MBP have a further approximate fourfold increase in* Bmp4* transcription over OPCs, but this response is downregulated upon maturation to myelinating oligodendrocytes (Figure 2). The obvious question arises: why is* Bmp4* transcription upregulated in OPCs, whose differentiation is significantly impaired by BMP4? One explanation is that increased local expression of BMP4 antagonists balances out this increased BMP4 expression by OPCs. Kondo and Raff observed increased noggin mRNA expression in both P6 optic nerve OPCs and astrocytes, but not oligodendrocytes [134]. Furthermore, experiments showing downregulation of BMP4 by WNT/*β*-catenin regulator Transcription Factor 7-like 2 (TCF7L) suggest that BMP4 expression by OPCs could be antagonised during development in a posttranscriptional manner to allow for oligodendrocyte differentiation [135]. This type of posttranscriptional regulation of BMP4 by specific glial progenitors requires further exploration.

However, as mentioned above, BMP4 can be expressed as a localised form tethered to the ECM as well as a secreted form. To date, no study has examined the exact isoform of BMP4 being expressed by OPCs. Another conjectural explanation for increased BMP4 expression in OPCs could be that local, ECM-tethered BMP4 acts as a spatial configuring mechanism during OPC differentiation to correctly space developing oligodendrocytes in the CNS. Oligodendrocytes can myelinate up to 50 individual axons in the CNS [136], and proper regional distribution of oligodendrocytes is likely crucial to maintaining appropriate and coordinated myelination. Perhaps BMP4 has a pragmatic role, preventing the differentiation of nearby OPCs and allowing newly developing oligodendrocyte progenitors to “stake out” a place nearby an unmyelinated neuron for subsequent myelination? Whether differential expression of localised and secreted forms of BMP4 in OPCs has functional relevance is an open and intriguing question.

## 7. BMP4 Signalling in SVZ NSCs and Neural/Glial Progenitors during Adulthood

**Figure 3:**
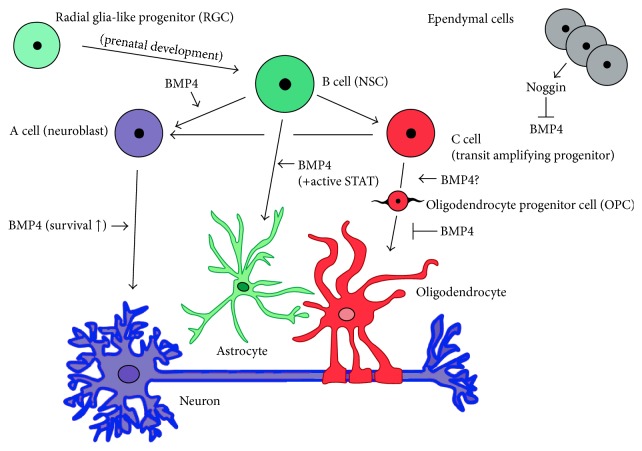
Simplified diagram of adult SVZ illustrating BMP4 involvement in NSC development. Adult NSCs (B cells) are specified from radial glia-like cells during prenatal development. Neuroblasts (A cells) and transient amplifying progenitors (B cells) are derived from NSCs and generate neurons and glia. Ependymal cells provide support by regulating CSF circulation and secrete BMP4 inhibiting noggin to modulate BMP signalling in the SVZ. BMP4 signalling through SMAD4 is important for neural specification of neuroblasts but does not influence further neuroblastic differentiation. It does appear to have a prosurvival effect on neuroblasts committed to the neuronal lineage. BMP4 signalling can promote astrogliogenesis from adult NSCs, but only with concomitant STAT-signalling, typically seen in CNS injury models. Recent evidence has shown that adult astrogliogenesis can occur from nestin+ SVZ NPCs, but the role of BMP4 in this process was not investigated. The role of BMP4 in OPC specification during development and adulthood is not completely resolved, but most data suggest that it does not play a significant role. However, there is a very clear inhibitory BMP4 effect on OPC progression towards an oligodendrocyte lineage during development, adulthood and CNS injury.

BMP4 continues to regulate NSC differentiation into neurons, astrocytes, and oligodendrocytes in the adult CNS. Given its role as a developmental regulator of NSC differentiation, it forms a crucial part of a larger signalling network maintaining an undifferentiated pool of NSCs and neural progenitors in the SVZ [10]. The cellular componentry of the SVZ consists of ependymal cells, and three classes of progenitor cells known as Type A, B, and C cells (see Figure 3). SVZ ependymal cells are nondividing support cells that facilitate cerebrospinal fluid circulation to the area [137] and can contribute to neurogenesis during stroke [138]. Type A cells are defined as chains of migrating neuroblasts and are generated from nearby highly proliferative Type C cells, which are known as transient amplifying progenitors (TAPs) or intermediate precursor cells (IPCs). Type B cells (or NSCs) are slow cycling progenitors with characteristics of astrocytes, but retaining stem-cell properties. These cells are further subdivided into B1 cells, which are located near the ependymal layer, and B2 cells, which associate closely with the adjacent striatal parenchyma [139].

BMP4 and its associated canonical receptors are expressed in both NSCs (B cells) and TAPs (C cells) in the adult SVZ [55, 140, 141]. SVZ ependymal cells also express noggin, which regulates levels of BMP4 signalling and modulates its neurogenic-gliogenic effects [55]. Colak et al. showed that deletion of SMAD1, a key downstream mediator of BMP4 signalling, severely impairs neurogenesis in the murine SVZ and acts early in the specification of NSCs to TAPs, which sequentially generate neuroblasts. Exogenous noggin infusion to the mouse SVZ promoted oligodendrogenesis over neurogenesis from TAPs. Phosphorylated SMAD1, SMAD5, and SMAD8 were also detected in SVZ GFAP+ cells and TAPs, but not in doublecortin (DCX)+ neuroblasts. The study did not specifically implicate BMP4 as a regulator of this effect but did note its increased expression and the presence of its canonical receptors and activated downstream SMADs. Interestingly, this study did note that BMP signalling in the SVZ does not promote astrogliogenesis. The authors speculated that, due to the lack of STAT expression in the SVZ [142], the induction of astrocytes via the BMP-dependent SMAD1/STAT interactions does not occur; thus, the neurogenic effect of BMP4 signalling predominates [143]. A recent study by Sohn et al. has demonstrated that corpus callosum and rostral migratory stream astrocytes are generated from SVZ nestin+ NPCs in mice; however, the role of BMP4/STAT signalling in this process has not yet been investigated [144]. Endogenous noggin expression likely allows for tight regulation of BMP concentrations in the SVZ to maintain progenitor pools. A similar result was also found in a study using chordin to modulate BMP4 levels and maintain progenitor cell plasticity in the SVZ [145].

## 8. BMP Signalling in Neural Stem and Precursor Cells Following CNS Injury

Given the role of the BMP4 in regulating maintenance and differentiation of NSCs during embryogenesis and adulthood, they represent a clear factor of interest in manipulation of endogenous and exogenous NSCs for therapeutic applications. The role of BMPs in CNS injury was comprehensively reviewed in a recent article by Grinspan [24]; this section will further examine key studies implicating BMP4 in the specification of NSCs and NPCs in CNS disease models.

Several CNS injuries have been shown to exhibit increased BMP4-SMAD signalling in neural stem cells and endogenous glial progenitors [146]. Given the well-characterised inhibitory effect of BMP4 on oligodendrocyte production, it has been extensively studied in the context of demyelinating disease [24, 129]. However, it has also been implicated in several other neurodegenerative and acute injuries of the CNS. BMP4 increases reactive astrogliosis* in vivo* [147] and is commonly upregulated by several cell types as a response to CNS injury [24]. It is unknown exactly what regulates this injury-induced upregulation of BMP4. Oxidative stress has been implicated in an intrauterine growth retardation (IUGR) model [148], but whether this is a common mechanism amongst other CNS injuries is currently unclear.

## 9. CNS Demyelination

BMP4 was first implicated in demyelinating disease through mRNA upregulation in demyelinated Multiple Sclerosis (MS) brain lesions [149]. Neural progenitors with a bipolar morphology and expression of polysialylated neuronal cell adhesion molecule (PSA-NCAM) have been identified as early oligodendroglial progenitors migrating from the SVZ during a demyelinating event [150]. Several experimental models of induced demyelination have demonstrated upregulated BMP4 signalling in both NSCs and OPCs. Increased generation of OPCs from NSCs has been shown to occur during focal demyelination localised near the SVZ [11, 151]. Interestingly, there is compelling evidence that pedigree matters during remyelination in the CNS. Xing et al. used genetic fate-mapping strategies during cuprizone-induced focal demyelination in the mouse corpus callosum to investigate the relative activities of NSC-derived OPCs and OPCs that migrate and differentiate from the brain parenchyma. From this, they showed that NSC-derived OPCs contribute to more extensive remyelination (measured by myelinated axon diameter) in the mouse corpus callosum after 6 weeks compared to parenchymal-derived OPCs [152]. As discussed above, BMP4 suppresses both NSC-derived oligodendrogliogenesis in the SVZ and adult OPC differentiation; thus, it may affect cell-mediated remyelination after demyelination at multiple levels in the CNS.

Ethidium bromide-induced demyelination causes a significant upregulation of BMP4 in mice. Zhao et al. observed that BMP4 mRNA was significantly upregulated in OPCs, whereas the expression of other BMPs, as well as noggin, did not change significantly [153]. The increased expression of BMP4 in OPCs upon commencement of remyelination (an endogenous repair response by local and migratory OPCs to a demyelinating insult) did not act in an autocrine manner as OPC differentiation during remyelination was not impeded. These data corroborate with subsequent RNA-Seq analysis of upregulated BMP4 transcription by postnatal OPCs [133] and further the intrigue of BMP4 expression in OPCs during adulthood and injury.

The effect of BMP4 in regulating NSC- and OPC-mediated CNS remyelination has also been investigated in two studies. Cate et al. showed that cuprizone-induced demyelination causes a significant upregulation of BMP4, its receptors BMPRIA, BMPRIB, and BMPRII, and phosphorylated SMAD1, SMAD5, and SMAD8 in the mouse SVZ [154]. Interestingly, in a follow-up study, BMP4 infusion during demyelination increased the numbers of proliferating OPCs [155]. However, increased generation of OPCs did not lead to increased numbers of oligodendrocytes, as has been shown in many studies assessing the differentiation block of OPCs in chronic demyelinating diseases [156]. Both studies showed that blocking BMP4 signalling via noggin infusion into the demyelinated areas of the mouse brain increased remyelination of damaged myelin sheaths.

As described above, during development, BMP4 is a crucial part of a complex signalling network involving WNT/*β*-catenin, FGF, Shh, and other major signalling pathways. As such, any attempt to modulate BMP4 signalling to ameliorate damage during and enhance repair after CNS injury must take into consideration possible crosstalk and regulation of associated pathways. For example, the inhibitory action of dysregulated WNT/*β*-catenin signalling on OPC differentiation has been demonstrated in demyelinating disease [157]. Feigenson et al. used* in vitro* OPC cultures to demonstrate that WNT/*β*-catenin signalling operates upstream of BMP4 signalling to mediate this effect. Both BMP4 and WNT/*β*-catenin signalling component Wnt3a inhibits oligodendrocyte differentiation in OPC cultures. Blocking of BMP4 via noggin application negated its astrogliogenic effect in OPC cultures despite the continued presence of Wnt3a, whereas inhibiting Wnt3a while retaining exogenous BMP4 treatment did not prevent increased astrogliogenesis [158]. They also demonstrated that Wnt3a does not promote astrogliogenesis from early postnatal OPC cultures derived from BMPRIA/B knockout mice. This relationship was further confirmed by a separate group* in vivo* using genetic knockout studies [135]. Genetic knockout of WNT/*β*-catenin effector TCF7l2, previously thought to suppress oligodendroglial differentiation by activation of WNT/*β*-catenin signalling, revealed that it has a dual role in inhibiting BMP4-mediated SMAD activation in OPCs and early oligodendrocytes. Whether this factor is relevant for NSC patterning and differentiation during embryonic development remains uncertain.

In addition to this, Wu et al. used an epigenetic approach to identify downstream histone deacetylation as a key transcriptional process regulated by BMP4 during CNS demyelination. BMP4 infusion during cuprizone-induced demyelination led to a significant increase in proliferating astrocyte numbers with elevated levels of acetylated histone H3 compared to vehicle-infused mice after 6 weeks. This was coupled with a decrease in mature oligodendrocytes and a transcriptional increase in downstream effectors for Notch and WNT/*β*-catenin such as* Hey1* and* Hes*. This agreed with their findings* in vitro* that BMP4 acts to suppress Shh-mediated histone deacetylation in OPCs. They postulated that, in OPCs, Shh-mediated histone deacetylases (HDACs) compacts chromatin and blocks access to promoters of astrocytic differentiation gene networks that are activated by convergence of BMP4, WNT/*β*-catenin, and Notch signalling [22]. Further investigation into common transcriptional elements between these related pathways is crucial for identifying optimal therapeutic targets for NSC/NPC-based regenerative therapies.

## 10. Spinal Cord and Other CNS Injuries and Disorders

BMP4 also has a defined role in regulating NPC differentiation in spinal cord injury (SCI). Wang et al. showed that conditioned media from astrocytes derived from the injured spinal cord of the mouse inhibited differentiation of OPCs into mature oligodendrocytes. Subsequent protein expression analysis indicated that BMP4 was upregulated in cultures of reactive astrocytes isolated from the injured spinal cord. Xiao et al. observed that BMP4 and phospho-SMAD1, phospho-SMAD5, and phospho-SMAD8 were upregulated in most neural cell types, including nestin+ NSCs, in response to induced SCI in mice. Predictably,* in vitro* spinal cord-derived NSCs pretreated with exogenous noggin prevented astrogliogenesis from subsequent BMP4 exposure. However,* in vivo* noggin application failed to completely suppress elevated GFAP+ expression in the injured spinal cord. The researchers attributed this to the continued activity of BMP4-independent promoters of astrogliogenesis including the CNTF/LIF-mediated JaK-STAT pathway [159].

Investigations into the formation of the astrocytic glial scar characteristic of spinal cord lesions have revealed intriguing functions for individual BMP Type I receptors. Sahni et al. conditionally deleted* Bmpr1a* and* Bmpr1b* from GFAP-expressing astrocytes prior to induction on SCI [160]. Injured wildtype animals displayed increased astrogliosis, increased phospho-SMAD1/SMAD5/SMAD8 expression by reactive astrocytes, as well as increased* Bmpr1a* transcript production. Injured* Bmpr1a* KO animals had reduced astrocytic hypertrophy compared to wildtype mice, leading to decreased astrogliosis around injured SC lesions, with increased immune cell infiltration as an indirect result. Surprisingly,* Bmpr1b* KO mice displayed an opposite reaction to SCI compared to the* Bmpr1a* mouse, with increased astrogliosis and accelerated wound closure, presumably from increased signalling through BMPRIA. Long-term, increased signalling through BMPRIB was found to attenuate glial scar progression and slow wound closure, leading to a poorer functional outcome compared to* Bmpr1a* KO mice. From this, it was suggested that signalling levels through the two receptors play opposing roles in modulating levels of astrocyte reactivity and subsequent glial scarring. While no differences in phosphorylated STAT3 or SMAD levels were observed in the* Bmpr1a* KO, differences in expression of microRNA-21 between* Bmpr1a* and* Bmpr1b* KO were suggested as a possible regulator of GFAP expression during SCI. This was further corroborated in a miRNA-21 overexpression system in mice [161].

Further work by North et al. showed that possible interactions of BMPRIB with *β*1-integrin at the cell membrane may alter the levels of downstream signalling activated by the receptor. *β*1-integrin is an ECM-interacting protein, a group of proteins that play an important role in stem cell maintenance. It was initially shown that *β*1-integrin expression was upregulated by ependymal zone cells in mice following SCI. Deleting *β*1-integrin in ependymal zone cell cultures, which generate nearly half of newly differentiated astrocytes following SCI, led to significant increases in GFAP expression and astrocytic differentiation compared to wildtype cells. Protein analysis of isolated lipid raft fractions from *β*1-integrin KO cultures identified an increased presence of BMPRIB. Subsequent disruption of lipid raft formation with a lipid raft inhibitor decreased both phospho-SMAD signalling and GFAP expression in these cultures. The researchers suggested that *β*1-integrin prevents the localisation of BMPRIB into lipid rafts, blocking further downstream signalling [162].

Experiments by Sandner et al. utilised bone marrow stromal cells (BMSCs) cotransplanted with NPCs to enhance repair after SCI in rats. The group had previously demonstrated that coculturing of BMSCs with hippocampal NPCs enhances the differentiation of the NPCs into mature oligodendrocytes via unknown secreted factors [163]. In this study, they demonstrated a similar effect* in vitro* using SVZ-derived NPCs. However, in the rat injured spinal cord, cotransplantation of BMSCs with SVZ-derived NPCs did not lead to enhanced oligodendrocyte numbers compared to animals receiving only NPCs. This differentiation block was linked to increased expression of BMPs 2 and 4 around the site of injury. Returning to* in vitro* culture assays, they showed that concurrent BMP2/4 treatment was sufficient to block the positive effect of BMSCs on oligodendrocyte differentiation from SVZ-derived NPCs.* In vitro*, they demonstrated that coculturing of NPCs with BMSCs overexpressing noggin via lentiviral gene transfer blocked the inhibitory effect of BMP4 [164]. Cotransplantation of noggin-overexpressing BMSCs combined with SVZ derived NPCs was not attempted but would be an interesting follow-up investigation. This study highlights a key challenge facing NSC transplantation for therapeutic applications: identifying and modulating critical intrinsic factors in the disease environment that may compromise the desired differentiation of newly transplanted cells.

Elevated BMP4 levels have also been observed in other CNS injuries and disorders including Alzheimer's disease [165] and intraventricular haemorrhage (IVH) in premature infants [166]. The latter study found BMP4 levels particularly elevated in the SVZ and in OPCs, suggesting a possible role in the hypomyelination displayed by IVH patients.

Clearly, the well-characterised astrogliogenic and antioligodendrogliogenic effect of BMP4 on glial progenitors can be problematic in CNS injuries, especially white matter injuries such as demyelination and SCI. As such, therapeutic targeting of BMP4 in CNS injury may be beneficial. However, the viability of BMP4-based therapeutics will depend on future research efforts to identify key components mediating the signalling pathway network. This will allow therapeutic approaches to effectively modulate specific undesirable BMP4 signalling outcomes without disrupting any of its potentially beneficial effects during the disease course.

## 11. Concluding Remarks and Future Perspectives

For a single protein, BMP4 has extensive influence on a multitude of CNS developmental and postnatal processes, as well as after CNS injury. BMP4 plays a part in the differentiation of NSCs into all three major classes of CNS cells: firstly neurons, then astrocytes, and all the while repressing oligodendrocyte lineage commitment throughout development and adulthood. Many questions have been addressed, but many remain: Does signalling through different receptor combinations produce different context-specific effects in NSCs? Is there extensive posttranscriptional regulation of BMP4 in NSCs and NPCs, adding further variation on an already dazzlingly complex regulatory network? To what extent does signal crosstalk between BMP4 and other signalling pathways regulate remyelination in the demyelinated CNS? Clearly, the BMP4 signalling pathway has extraordinary breadth of activity in regulating NSC and NPC biology during development, adulthood, and disease. Fully expounding its intricacies and relationships with other signalling pathways will be beneficial for further therapeutic application of NSCs and NPCs.

## Abbreviations

NSC: Neural stem cell
NPC: Neural progenitor cell
BMP: Bone morphogenetic protein
SVZ: Subventricular zone
SGZ: Subgranular zone
OPC: Oligodendrocyte progenitor cell
GFAP: Glial fibrillary acidic protein
MH: Mad homology
Shh: Sonic hedgehog
FGF: Fibroblast growth factor
MAPK: Mitogen-activated protein kinase
TGF-*β*: Transforming growth factor *β*
EGFR: Epidermal growth factor receptor
ERK: Extracellular signal-related kinase
BMPR: Bone morphogenetic protein receptor
ECM: Extracellular matrix
bHLH: Basic helix-loop-helix
RGMa: Repulsive guidance molecule A
PAWS1: Protein associated with SMAD1
SMURF: SMAD Ubiquitin Regulatory Factor (SMAD specific E3 ubiquitin protein ligase)
PDGFR*α*: Platelet-derived growth factor receptor-*α*
TAPs: Transient amplifying progenitors
IPCs: Intermediate precursor cells.
